# Tackling the Disproportionate Burden of Resistant Hypertension in US Black Adults

**DOI:** 10.1007/s11886-024-02115-5

**Published:** 2024-09-05

**Authors:** Tina K. Reddy, Samar A. Nasser, Anuhya V. Pulapaka, Constance M. Gistand, Keith C. Ferdinand

**Affiliations:** 1 Department of Medicine, Johns Hopkins University School of Medicine, Baltimore, MD USA; 2 Department of Clinical Research & Leadership, School of Medicine and Health Sciences, The George Washington University, Washington, DC USA; 3 Department of Medicine, Tulane University School of Medicine, New Orleans, LA USA

**Keywords:** Resistant hypertension, Black patients, Disparities, Implementation

## Abstract

**Purpose of Review:**

Elevated blood pressure is the leading modifiable risk factor for cardiovascular morbidity and mortality in the US. Older individuals, Black adults, and those with comorbidities such as chronic kidney disease, have higher levels of uncontrolled and resistant hypertension. This review focuses on resistant hypertension, specifically in the US Black population, including potential benefits and limitations of current and investigational agents to address the disparate toll.

**Recent Findings:**

There is a necessity to implement public health measures, including early screening, detection, and evidence-based hypertension treatment with lifestyle, approved and investigational agents. The evidence highlights the importance of implementing feasible and cost-effective public health measures to advocate for early screening, detection, and appropriate treatment of hypertension.

**Summary:**

A team-based approach involving physicians, advanced practice nurses, physician assistants, pharmacists, social workers, and clinic staff to implement proven approaches and the delivery of care within trusted community settings may mitigate existing disparities.

## Introduction

Globally, hypertension (HTN) prevalence is greater than 1.5 billion, and high blood pressure (HBP) is the leading modifiable risk factor attributable to premature cardiovascular (CV) deaths [1]. In 2021, HBP was associated with ischemic heart disease and stroke-related deaths, accounting for 10.8 million (95% CI: 9.15–12.1 million) CV deaths and 11.3 million (95% CI: 9.59–12.7 million) deaths overall [2]. Paradoxically, the United States (US) has the lowest life expectancy among large wealthy countries, yet far outspends similarly matched nations on health care. With the mean US life expectancy of 76.1 years, much less than in Western Europe or Japan, the health care spending estimated in 2021 of 12,318 US dollars per capita was more than twice that of comparable countries. Hypertension affects ~ 121.5 million US adults, nearly half of this population [3]. Moreover, effective blood pressure (BP) control prevents incident heart failure (HF) [2]. Unfortunately, there is a looming tsunami of cardiovascular disease (CVD) morbidity and mortality in the US, driven by HTN and the burden of other uncontrolled risk factors [4]. In addition, older individuals, Black adults, and those with comorbidities such as diabetes, chronic kidney disease (CKD), and CVD appear to be more susceptible to uncontrolled HTN. Moreover, given the expanding aging population, uncontrolled HTN prevalence is rising, and effective interventions are required to thwart this public health crisis. The HTN prevalence in Black/African American individuals is among the highest in the world and according to the 2017 to 2020 National Health and Nutrition Examination Survey, the age-adjusted prevalence of HTN among non-Hispanic Black (NHB) male adults was 55.8% and 56.9% among females [3, 5]. Furthermore, older individuals, Black adults, and those with comorbidities such as diabetes, CKD, and CVD appear to be more susceptible to asymptomatic elevated inpatient BP or hypertensive emergencies [6]. Additionally, the social determinants of health (SDOH), including healthcare access or living in low-income areas, increases the risk of hospitalization for HTN [6].

Contemporary clinical trial data in middle-aged and older adults confirm the association between intensive BP lowering and reduced CV morbidity and mortality, potentially extending life expectancy by up to 3 years [7–9]. Unfortunately, there has been a downward trend in overall US life expectancy, driven primarily by increasing CVD and widened by COVID-19, with a persistent mortality gap between White and Black individuals [3, 10]. Therefore, control of HTN and other risk factors is the most cost-effective means to reversing the rising CVD mortality and the persistence of unacceptable racial/ethnic disparities.

## Disparities in Hypertension Impacting US Black Populations

Despite similar levels of HTN awareness and treatment, compared to the White population, control rates are disparate in NHB, Hispanic, and Asian American populations [3]. As a result, HTN is the leading cause of HF-related mortality in Black populations, particularly among younger adults aged 15–44 years, with an overall attributable population risk 4.3 times higher (95% CI 2.3 – 6.3) [11]. Furthermore, uncontrolled HTN and diabetes leads to a higher burden of end stage renal disease (ESRD), especially among Black adults. However, this increase in CKD in Black adults may be impacted by a greater prevalence of high-risk variants in the apolipoprotein L1 gene, termed G1/G2 [12]. Alarmingly, although the Black population is only 13.6% of the US population, the percentage of persons with ESRD was 31.6% in 2021, with a prevalence over 4 times that of White individuals [13].

Overall, to reduce the burden of HTN and CVD in Black populations, SDOH must be addressed, including inadequate health care access, low socioeconomic status, limited educational attainment, lack of safe and affordable housing, insufficient transportation options, low social support, limited healthy food availability, structural inequities, and intrinsic bias. Factors which should be minimized include simply reporting skin color or self-identified race and suggesting unmeasured but assumed genetic factors [14]. The SDOH (where people work, eat, live, and pray) have a profound effect on life expectancy [15]. Accordingly, the recent American Heart Association (AHA) Predicting Risk of CVD EVENT (PREVENT) risk model for primary prevention goes beyond conventional well-defined risk factors to include the SDOH defined by the social determinant indicators by zip code, hemoglobin A1c, and urine albumin: creatinine ratio. This new approach may inform the need and intensity for BP reduction pharmacotherapy [16].

This review focuses on evidence-based current approaches, as well as investigational medications, renal nerve intervention, and bariatric surgery, to potentially address the disparate toll of resistant HTN in the Black population, recognizing the well-demonstrated direct, linear, and persistent relationship between intensity of BP reduction and prevention of major CVD events [17]. A shortcoming of well-executed large evidence-based trials, such as the Systolic Blood Pressure Intervention Trial (SPRINT) (N = 2944), as highlighted in a subset evaluated after 10 years, is that the benefits of a rigorous intensive care research model may be lost over time [18]. Most adult patients require two or more antihypertensive medications to achieve a BP of less than 130/80 mm Hg, and this is particularly true for Black adults [19]. Clinicians must be aware of adverse SDOH, especially for financially disadvantaged patients and facilitate access to fixed-dosed combination anti-hypertensive medications to improve adherence and BP control [20].

## Focus on the Impact of Resistant Hypertension

Currently, resistant HTN is defined as uncontrolled BP (≥ 130 mm Hg SBP and/or ≥ 80 mm Hg diastolic BP), despite the concurrent use of 3 antihypertensive drug classes at maximally tolerated doses including a thiazide diuretic. Furthermore, resistant HTN is controlled BP if requiring ≥ 4 antihypertensive medications [21]. Additionally, secondary causes of HTN, white coat effect, and medication non-adherence must be excluded. Overall, HTN including resistant HTN, has led to decreased life expectancy and increased CV morbidity and mortality in NHB populations. Multilevel efforts will be needed to resolve race-related CV disparities at the patient, physician, community, and health system levels [21, 22]. Controlling BP and other CV risks will be essential to reducing disparities. However, achieving health equity has been difficult. Without targeted interventions to identify and eliminate disparities, the persistent unequal outcomes in the US population will continue. Specific targeted interventions are needed to overcome disparities not only by race/ethnicity, but also by sex/gender, geography, socioeconomic status, ability or disability. Addressing these inequities is not only a moral, but also a practical imperative [23].

**Fig. 1:**
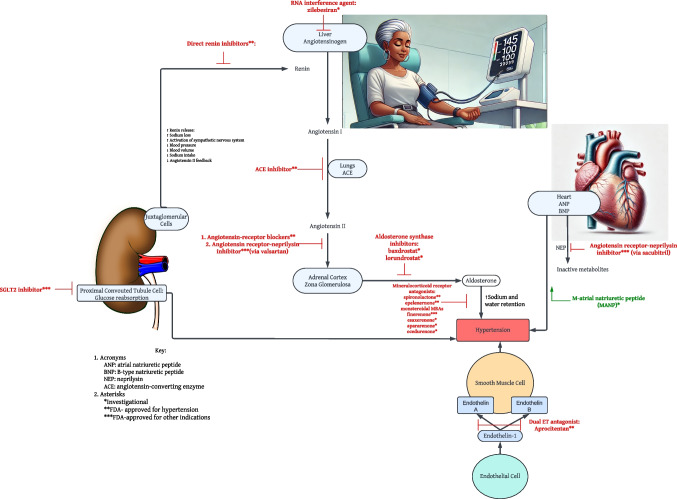
Blood Pressure Lowering Mechanisms for Current and Emerging Agents

Specifically, approaches to resistant HTN must include lifestyle modifications as the cornerstone of therapy, including a low sodium diet (< 2400 mg/d), ≥ 6 h of uninterrupted sleep, healthy dietary patterns, weight loss, and physical activity [24]. Optimal therapy prior to making the diagnosis of resistant HTN includes three anti-hypertensive classes at maximum or maximally tolerated doses: renin-angiotensin system (RAS) blockers, calcium channel blockers, and diuretics. Specifically, in Black adults with HTN, when a renin-angiotensin system modulator is required, an angiotensin receptor blocker (ARB) may be preferred over an angiotensin-converting enzyme (ACE) inhibitor. There are data reflecting a small increased risk of angioneurotic edema with ACE inhibitors in people of African descent. In such patients, an ARB may be a preferred RAS blocker [25]. The diuretic type and dosages should be appropriate for renal function, with chlorthalidone or indapamide being possible options. In an additional step, a mineralocorticoid receptor antagonist (MRA), either spironolactone or eplerenone may be added. Although eplerenone is less effective on a mg-to-mg basis, it avoids the off-target effects, particularly breast tenderness, gynecomastia, and sexual dysfunction [24]. Along with the approved agents, there are investigational agents which are being developed which may assist with control in patients with difficult-to-treat and resistant HTN. Furthermore, agents which have been FDA approved for other indications, such as SGLT2 inhibitors, the angiotensin receptor/neprilysin inhibitor (ARNI), and finerenone may benefit these high-risk patients (Fig. 1).

Nevertheless, the primary reason patients have resistant HTN is undertreatment. Specifically, for Black adults with prior treatment resistant HTN, data from the Jackson Heart Study and the Reasons for Geographic and Racial Differences in Stroke study (1,776 participants and 65.9% female) demonstrated only 1.2% had ideal lifestyle factors. Moreover, only 5.9% were taking chlorthalidone or indapamide, despite the recommended benefits, and less than 10% (9.8%) were taking spironolactone or eplerenone. This suggests that evidence-based lifestyle factors and recommended pharmacological treatments are underutilized in Black adults with apparent treatment-resistant HTN [26].

## Novel Patient-Centered Interventions for Hypertension Control in Diverse Populations

Novel approaches include community-based interventions such as the Healthy Heart Community Prevention Model in New Orleans, LA, which was first initiated and conceived as a 1-year pilot program funded by the National Heart, Lung, and Blood Institute of the National Institutes of Health as an outgrowth of the National Medical Association’s Healthy People 2000 program [27]. Patient-centered approaches include the attempt to promote and educate on CV health by utilizing barbershops, salons, and faith settings. The community-based approach was successfully implemented in a rigorous hypertensive study by the late Ronald Victor and others [28]. Although not yet definitively proven, improving BP, medication adherence, and health care utilization by promoting self-measured BP may be critically important [29]. Moreover, a pilot study in New Orleans, LA, Text My BP Meds NOLA, demonstrated that text-messaging and social support benefited 36 patients (63.9% female, 88.9% NHB patients​) with significant systolic BP reduction from baseline (142.19 ± 18.94 to 131.69 ± 13.99, p-value = 0.0027) [30]. These patients had multiple risk factors, including a body mass index (BMI) of 34.8 ± 7.9​, 74.3% with diabetes mellitus (DM)​ and 72% with obesity. Potentially, the implementation of text-messaging and social support interventions will improve HTN management among traditionally underserved, high-risk patients [30]. Furthermore, a recent systematic review and meta-analysis confirmed the benefit of digital HTN health interventions, including in NHB populations. Among the 8,257 patients across 28 studies, systolic BP decreased by 4.24 mm Hg at 6 months (95% CI, − 7.33 to − 1.14 mm Hg; P = 0.01) [31]. Another systematic review and meta-analysis of church-based interventions (n = 23 studies) to control risk factors suggested an overall improvement in body weight by -3.1 lb, [95% CI, -5.8, -1.2], N = 15), waist circumference by -0.8 in, [CI, -1.4, -0.1], N = 6)​, and systolic BP by -2.3 mm Hg, [CI, -4.3, -0.3], N = 13) [32].

Dissemination of community-based interventions have been initiated such as the **C**hurch-based **H**ealth Intervention to **E**liminate **R**acial **I**nequalitie**s** in Cardiovascular **H**ealth (CHERISH)​ in New Orleans [33]. Integration of key strategies to improve BP control have recently been published by the AHA and include accurate BP measurement, use of team-based care, implementation of lifestyle programs at organizational, community, and policy levels; use of standardized treatment protocols; improved medication acceptance and adherence; use of continuous quality improvement; use of financial levers; and use of antiracism efforts [34]. Most recently, a 24-month randomized clinical trial (RCT) was conducted in Valencia, Spain demonstrated the long-term effectiveness of BP self-monitoring plus self-titration of BP medication [35]. In the future, simple and inexpensive self-management interventions may ameliorate the public health impact of uncontrolled HTN, including in the US Black population.

In summary, addressing resistant HTN in the Black American community involves multilevel efforts. Beneficial effects may include several positive components: a collaborative agreement between physicians and team-based care, effective anti-hypertensive drug regimens, and trusted community sites for BP intervention [36]. Ultimately, shared decision-making and empowering patients to participate in their care should remain the cornerstone for providers to successfully implement and disseminate multi-level approaches to care.

## Current and Emerging Resistant Hypertension Treatments

Resistant HTN portends a greater risk for adverse CV outcomes when compared to controlled HTN, with prevalence widely ranging from 4.3% to 29.7% [37]. In a recent evaluation of electronic health record (EHR) databases from ambulatory patients with HTN, the prevalence of apparent treatment resistant HTN was 11.3–16.7%. On further analysis, the data demonstrated that similar significant predictors in both EHR populations included Black race, diabetes, HF, CKD, cardiomegaly, and higher BMI. Notably, Black race was the strongest predictor in both populations [37].

Several novel therapies are being investigated for the treatment of resistant HTN with at least one newer agent recently approved by the US Food and Drug Administration (FDA). Moreover, at least three agents approved for other clinical indications may have a role in BP-lowering for resistant HTN (Table 1).

**Table 1:** New and Emerging Agents for Resistant Hypertension with the Mechanism of Action

M-atrial natriuretic peptide (MANP)*: Enhances natriuretic, aldosterone-suppressing actions with a novel ANP analog
Endothelin receptor antagonist (ERA)**—FDA approved: Reduces blood endothelin levels and inhibits binding to receptors in smooth muscles cells, leading to vasodilation
Angiotensinogen interference agent*: Reduces hepatic angiotensinogen via messenger RNA levels to lower angiotensinogen production
Aldosterone synthase inhibitors*: Inhibits aldosterone production, reducing excess sodium and water reabsorption
Nonsteroidal mineralocorticoid receptor antagonists (MRA)*: Blocks MR, preventing aldosterone activation, reducing sodium and water reabsorption (finerenone*** approved for CKD and CVD with T2DM)
Dual angiotensin II receptor-neprilysin inhibitor (ARNI)***—FDA approved for HF: Blocks neprilysin, preventing natriuretic peptide breakdown, reducing BP, sympathetic tone, and aldosterone levels, combined with angiotensin receptor blocker
Sodium-glucose cotransporter 2 inhibitors (SGLT2i)***—FDA approved for T2DM, CKD, HF: Reduces renal reabsorption of glucose and sodium, exerting hypoglycemic and antihypertensive effects, improves endothelial function, and reduces arterial stiffness

^*^Investigational – not FDA approved for any therapy

^**^FDA-approved for hypertension

^***^ Approved, but for other FDA indications

BP: blood pressure; HTN: hypertension; ANP: Atrial Natriuretic Peptide; CKD: chronic kidney disease HF: heart failure; MR: mineralocorticoid receptor; Ang I: Angiotensin I; Ang II: Angiotensin II

Early reports of an investigational M-atrial natriuretic peptide (MANP) with HTN and metabolic syndrome demonstrated a meaningful BP reduction of approximately 5–10 mm Hg. Additionally, MANP exhibited beneficial effects on CV and metabolic health, also increasing cyclic guanosine monophosphate and non-esterified fatty acid, decreasing glucose, and improving insulin resistance [38].

In the Jackson Heart Study (JHS), higher endothelin was associated with higher risks of BP progression (an increase by ≥ 1 BP category based on the 2017 American College of Cardiology/American Heart Association classification) and the development of HTN [38]. Much of the attention on new investigational agents focus on the beneficial effects of aldosterone synthase inhibitors and blockade of endothelin A and B receptors. The agents significantly reduce BP in patients with low renin HTN, volume overload, salt sensitivity, endothelial dysfunction, and arterial stiffening, many of which are prevalent in Black patients. In patients with treatment resistant HTN, this approach may lead to correction of HTN and reduction of target organ damage, although this has not been shown in prospective RCTs.

Despite a pathophysiologic link between endothelin-1 and HTN in Black adults, in a NHB cohort (N = 1197, mean age 47.8 years, 64.2% women), endothelin-1 concentrations were associated with a higher risk of BP progression and incident HTN [39]. Accordingly, Schlaich and colleagues demonstrated the benefit of a dual endothelin antagonist, aprocitentan, in patients with resistant HTN in a multi-center, blinded, parallel group, phase 3 trial [40]. Patients (mean age of 62 years, 60% male, 83% identified as White, 11% African American, 5% Asian, approximately 10% Hispanic, mean BMI of 34) showed significant reductions in both daytime and nighttime BP, including in the Black cohort with aprocitentan, with mild to moderate edema especially seen at the higher dose of 25 mg [40]. More recently, the FDA approved (3/2024) the use of 12.5 mg aprocitentan for the treatment of HTN, in combination with other anti-hypertensive drugs, to lower BP in patients who are not adequately controlled [41]. Despite the lack of a specific outcome study, the FDA noted that lowering BP reduces the risk of fatal and non-fatal CV events—primarily stroke and MI. In reported data, aprocitentan was well tolerated, with clinically relevant reductions in systolic BP documented by ABPM (ambulatory blood pressure monitoring) in Black patients [42].

A recent review of the effects of the renin–angiotensin–aldosterone system and various pharmacotherapeutic agents outlined potential targets that are receiving increasing attention and undergoing more research [43]. Recently, RNA-interfering agents for angiotensinogen, such as zilebesiran, have targeted the first step of the renin-angiotensin system. The RNA interference approach with zilebesiran was demonstrated in patients with mild to moderate HTN in a randomized trial (N = 394​, 24.5% Black patients​, 44.3% women​, with a mean age 57 years) with significant changes in SBP from baseline to 3 months compared to placebo [44]. Additionally, zilebesiran in combination with standard-of-care antihypertensive medications showed beneficial BP reduction in patients with inadequately controlled HTN. The study included a significant proportion of Black participants in the treatment groups: 23.6% on indapamide (N = 127), 33.5% on amlodipine (N = 239), and 25.6% on olmesartan (N = 301) [45]. Zilebesiran appeared to significantly lower BP to -12 for indapamide (-16.5, -7.6, p < 0.001), -9.7 for amlodipine (-12.9, -6.6, p < 0.001), and -4.0 for zilebesiran (-7.6, -0.3, p = 0.036). Targeting the first step of the renin-angiotensin system appeared to effectively and safely lower BP without any major adverse effects [45].

Recent data from the JHS confirmed the association between higher levels of serum aldosterone and lower plasma renin activity on ambulatory BP in Black patients. The plasma renin activity (PRA) was negatively associated and the aldosterone:renin ratio was positively associated with clinic, awake, and asleep systolic and diastolic BP and several ABPM phenotypes. In this population, renin suppression appears to be a critical factor underlying higher degrees of HTN and a target for pharmacotherapy. A suppressed renin phenotype (PRA ≤ 0.50 ng/ml/hr) was associated with higher BPs and odds of multiple ABPM phenotypes compared to unsuppressed renin phenotype (PRA ≥ 1.0 ng/ml/hr) [46]. Thus, further research on MRA or epithelial sodium channel inhibitors may represent innovative therapeutic targets to reduce health disparities in Black patients with HTN. In US Black adults, low potassium intake may be a significant factor in increasing BP, and supplementation has been shown to improve HTN and renal hemodynamics in African American patients. This supports the concept that low potassium intake may play a role in some of the physiologic indices in this population and increasing potassium may assist with BP reduction [47].

Given the importance of increased aldosterone activity as a hallmark of resistant HTN, particularly in NHB adults, aldosterone synthase inhibitors may be a valuable addition to the pharmacotherapeutic agents for managing resistant HTN. Newer agents, such as baxdrostat and lorundrostat, have also shown potential effectiveness as aldosterone synthase inhibitors​. Phase 2 data on baxdrostat demonstrated dose-dependent reductions in systolic BP with no serious adverse events. Baxdrostat at 1 and 2 mg dosages lowered SBP by -17.5 (p = 0.003) and -20.3 (p < 0.001), respectively, compared to -9.4 with placebo [48]. In a recent phase 2 trial for treatment-resistant HTN, Laffin and colleagues demonstrated the efficacy of lorundrostat in a randomized, placebo-controlled, dose-ranging trial among adults with uncontrolled HTN on two or more anti-HTN medications [49]. Among the 163 participants, mean age was 65.7 (SD, 10.2) years, 60% were women, 36% were Black ethnicity. Those receiving twice-daily doses of 25 mg and 12.5 mg of lorundrostat had systolic BP decreases of − 10.1 and − 13.8 mm Hg, respectively. Overall, lorundrostat has a favorable safety profile and significant BP reduction, specifically among those with HTN and obesity.

Building on the effective addition of MRA agents such as spironolactone, emerging non-steroidal MRA therapies are promising (finerenone, esaxerenone, apararenone and ocedurenone). However, as newer agents are developed for the treatment of resistant HTN, their use may be inadequate to reduce disparities since access and cost remain significant barriers. Finerenone​ has been approved in the US for the control of diabetes with CKD, while others may hold the promise of even more effective BP control [43]. An advantage of these non-steroidal MRAs is that they are selective and have less affinity for the steroid hormone receptor, resulting in fewer adverse effects such as low libido, gynecomastia, and impotence compared to MRAs [50, 51]. Accordingly, a subgroup analysis of ocedurenone, conducted as part of a phase 2b, placebo-controlled study, included participants with a mean age of 65.4 years, 45.1% were female, although the majority (92%) were White patients and 21.6% self-identified Hispanic patients. In this particular population, 64 (39.5%) had stage 4 CKD, 51 (31.5%) had DM, 125 (77.2%) had albuminuria (urinary albumin-creatinine ratio ≥ 30 mg/g), and 85 (52.5%) had very high albuminuria [52]. Although approved for clinical use in Japan, this study suggests that ocedurenone may be effective across multiple subgroups, including Hispanic patients and those with CKD Stage 4, diabetes, and very high albuminuria.

Three classes of drugs are not approved for HTN, but may be used for other CVD treatments, including finerenone, sacubitril/valsartan, and sodium-glucose co-transporter 2 inhibitors (SGLT2i) and have been shown to lower BP. Currently under investigation for resistant HTN, XXB-750 is an experimental injected drug that targets natriuretic peptide (NPR1). A global Phase 2 study comparing XXB-750 to placebo is ongoing to evaluate efficacy, safety, and tolerability among patients with resistant HTN [53].

Beyond broad benefits in CV morbidity in patients with HF, CKD, and diabetes, SGLT2i agents have the potential beyond cardiac-specific therapy [54, 55]. Accordingly, the antihyperglycemic and BP effects of empagliflozin (an SGLT2i) in Black patients with Type 2 DM and HTN were demonstrated in a study of 150 patients using 24-h ambulatory BP [56]. The cohort was 52.7% male with a mean age of 56.8 (9.3) years and a mean duration of type 2 DM of 9.3 years. At week 24, empagliflozin at doses of 10–25 mg demonstrated a -10.33 mm Hg adjusted mean change in ambulatory systolic BP compared to placebo (-1.94 mmHg). Although SGLT2i are not utilized for HTN per se, these medications may provide additional HTN benefit in higher risk patients [56].

## Renal Nerve Denervation and Bariatric Surgery for Resistant Hypertension

Recently, the FDA approved two renal denervation (RDN) systems for the treatment of HTN: the Paradise Ultrasound Catheter Water-cooled balloon (RADIANCE-HTN SOLO) [FDA Approved Nov 7, 2023] and Symplicity Spyral Catheter: Radiofrequency energy [FDA Approved November 19, 2023] [57, 58]. A third approach, the Peregrine system alcohol infusion catheter, which injects ethanol into the periarterial tissue adventitial space is not yet approved [59, 60].

In the Paradise system FDA label, Black patients comprised ~ 20% of the patient populations and no safety or efficacy-related differences were observed compared to the overall patient population [58].

In data involving the Spyral catheter from the US sham cohort, the treatment effect may have been diluted, particularly among Black Americans [61]. In patients with RDN, the sham reduction in the Black cohort (N = 22) was -26.2 vs -16.4 in the RDN group (p-value 0.200). In those taking at least one medication three or more times a day in the sham group, there was an increase in medication adherence during the trial. This phenomenon may be due to the distrust and mistrust of healthcare systems within Black American communities, along with nonadherence. Essentially, when a person is enrolled in a clinical trial and has more intense shared decision-making and attention to the risk of elevated BP, improved BP in the sham control may have blunted any outcome differences with RDN.

In consideration of the impact of obesity in HTN, a recent study documented that bariatric surgery may be effective not only for weight loss but also for BP. Roux-en-Y gastric bypass plus medical therapy demonstrated a significant antihypertensive effect among 100 participants after 5 years. Most patients were female (76%), mean age was 43.8 ± 9.2 years, and BMI of 36.9 ± 2.7 kg/m^2^. Overall, 80.7% of patients reduced their antihypertensive medications by at least 30%, with a BP of less than 140/90 mm Hg, and 46.9% achieving remission. Thus, obesity effectively treated with surgery in high-risk patients with HTN demonstrated an additional benefit for BP control [62].

## Conclusions

There is a pressing need to address ongoing racial and ethnic disparities in CVD health in the US, with a persistent mortality gap, specifically between White and Black individuals. This mortality disparity is primarily driven by CVD and increased health care costs, threatening the promise of the US as an egalitarian society. Control of HTN will be one of the best means of addressing racial inequities, as resistant HTN is more prevalent and persistent in Black populations. Proposed strategies to improve BP control include: increasing the accuracy of measurements, emphasizing team-based care that promotes healthy lifestyles, and standardizing treatment protocols to improve medication acceptance and adherence [22]. Implementation of evidence-based medicine includes the use of current guideline-directed care and the potential utilization of new investigational therapeutic agents, renal nerve intervention, and bariatric surgery to address uncontrolled HTN.

## Data Availability

No datasets were generated or analysed during the current study.
